# Updated Bayesian network meta-analysis on the efficacy and safety of PD−1 versus PD−L1 inhibitors in first−line treatment with chemotherapy for extensive−stage small-cell lung cancer

**DOI:** 10.3389/fonc.2024.1455306

**Published:** 2025-01-28

**Authors:** Ke Wang, Chuangjie Zheng, Xinrong Chen, Penghui Lin, Mengge Lin, Cuizhen Chen, Linzhu Zhai

**Affiliations:** ^1^ Guangzhou University of Chinese Medicine, Guangzhou, China; ^2^ Lingnan Medical Research Center, Guangzhou University of Chinese Medicine, Guangzhou, China; ^3^ Cancer Center, Departments of Radiation Oncology, the First Affiliated Hospital of Guangzhou University of Chinese Medicine, Guangzhou, China

**Keywords:** extensive-stage small-cell lung cancer, efficacy and safety, PD-1 inhibitors, PD-L1 inhibitors, network meta-analysis

## Abstract

**Objective:**

To compare the efficacy and safety of programmed cell death 1 inhibitors plus chemotherapy (PD-1 + Chemo) and programmed cell death ligand 1 inhibitors plus chemotherapy (PD-L1 + Chemo) for the treatment of extensive-stage small-cell lung cancer (ES-SCLC).

**Methods:**

We performed a meta-analysis of relevant data using R software, considering overall survival (OS), progression-free survival (PFS), and grade ≥ 3 treatment-related adverse events (TRAES).

**Results:**

PD-1 + Chemo (OS: hazard ratio [HR] 0.71; PFS: HR 0.59) and PD-L1 + Chemo (OS: HR 0.72; PFS: HR 0.73) significantly prolonged survival and did not increase the incidence of grade ≥3 TRAEs compared with chemotherapy. Indirect comparisons showed no significant difference in clinical efficacy (OS: HR 0.99, 95% CI: 0.86–1.1; PFS: HR 0.80, 95% CI: 0.61–1.0) or safety (HR 1.0, 95% CI: 0.93–1.1) between PD-1 + Chemo and PD-L1 + Chemo. Non-cumulative probability ranking plot ranking results showed that PD-1 + Chemo ranked first in OS and PFS. Patients with PD-L1 expression levels < 1%, PD-1 + Chemo showed a trend of disadvantage (OS: HR 1.3; PFS: HR 1.2), whereas for patients with PD-L1 expression levels ≥ 1%, PD-1 + Chemo showed a trend of advantage (OS: HR 0.85; PFS: HR 0.85).

**Conclusions:**

PD-1 + Chemo and PD-L1 + Chemo significantly prolonged OS and PFS in patients with ES-SCLC and did not significantly increase the incidence of grade ≥ 3 TRAES. The efficacy and safety profiles of PD-1 + Chemo and PD-L1 + Chemo appear to be similar.

## Introduction

Small-cell lung cancer (SCLC) is a poorly differentiated neuroendocrine tumor with low differentiation, high malignancy, rapid growth, and early widespread metastasis, and it accounts for about 13%–17% of all lung cancers (1, 2). According to the Veterans’ Administration Lung Study Group staging system, about 70% of patients have already entered extensive-stage SCLC (ES-SCLC) at the time of the initial diagnosis. ES-SCLC has a poor prognosis, with a 5-year survival rate of no more than 5%, and the average overall survival (OS) of patients without systemic treatment is unlikely to exceed 4 months (2). Platinum-based chemotherapy has been the standard first-line regimen for the treatment of ES-SCLC in the past, and the median survival rate for patients with ES-SCLC receiving a platinum (cisplatin or carboplatin) combined with etoposide (EP) chemotherapy regimen is only 9–11 months (3). This therapeutic dilemma remained unbroken for many years until immune checkpoint inhibitors (ICIs) brought new hope to ES-SCLC patients with superior survival rates.

Of the large prospective studies of immunotherapy in ES-SCLC, the results of the IMpower 133 and CASPIAN studies were the first to break the logjam in first-line treatment of ES-SCLC, significantly improving patients’ survival rates (4, 5). Subsequently, two large prospective clinical studies, ASTRUM-005 and CAPSTONE-1, supported the use of serplulimab and adebrelimab in the first-line treatment of ES-SCLC patients (6, 7). In 2023, results from the RATIONALE-312 study showed that the programmed cell death-1 (PD-1) inhibitor tislelizumab in combination with chemotherapy prolonged the median OS of patients to 15.5 months, thus achieving a significant improvement in survival in patients with ES-SCLC (8). The results of the EXTENTORCH study also showed a significant improvement in OS and a higher 1-year OS rate in the toripalimab-combination chemotherapy group compared with the chemotherapy-only group (9). Currently, only three PD-1 inhibitors and three PD-L1inhibitors have achieved positive results in Phase III studies in SCLC. Based on the success of immunotherapy in the treatment of SCLC, PD-1/PD-L1 inhibitors in combination with chemotherapy have become the new standard first-line treatment option for ES-SCLC (10).

In recent years, several meta-analyses have assessed the difference in efficacy and safety of PD-1/PD-L1 inhibitors in combination with chemotherapy in ES-SCLC (11–13). However, the results of all of the trials included in these meta-analyses were published before October 8, 2023. With the results of two recent studies, RATIONALE-312 and EXTENTORCH, the data on PD-1 inhibitors in the treatment of ES-SCLC have been further enriched. Thus, there is an urgent need for updated meta-analyses on the efficacy and safety of PD-1 and PD-L1 inhibitors in patients with ES-SCLC to inform clinical practice.

## Methods

### Data source and search strategy

Computerized searches of PubMed, Embase, and Web of Science were performed, and to include the most recent findings, we also searched the online proceedings of the annual meetings of the American Society of Clinical Oncology, Chinese Society of Clinical Oncology, European Society for Medical Oncology, and the World Congress of Lung Cancer. The search deadline to extract randomized controlled trials (RCTs) on the efficacy and safety of PD-1/PD-L1 inhibitors in combination with chemotherapy versus chemotherapy alone for ES-SCLC was November 8, 2023.

### Inclusion and exclusion criteria

Inclusion criteria were: (1) SCLC patients with histopathological and/or cytological confirmation; (2) RCTs comparing the efficacy and safety of PD-1/PD-L1 inhibitor combination chemotherapy versus chemotherapy alone in the treatment of ES-SCLC; (3) RCTS where the experimental group was treated with PD-1/PD-L1 inhibitor combination chemotherapy, and the control group received chemotherapy alone; and (4) the outcome indicators were OS, progression-free survival (PFS), and the incidence of grade ≥ 3 treatment-related adverse events (TRAEs) associated with treatment.

Exclusion criteria were: (1) RCTs that were based on overlapping patients; (2) no available outcome metrics; and (3) data with obvious errors or unavailable data that could not be extracted after contacting the authors.

### Data extraction and quality assessment

Literature was screened, data extracted and cross-checked independently by two researchers, and any discrepancies were resolved by discussions with a third party. Information extracted included trial name, year of publication, authors, trial period, sample size, age, sex, national clinical trial identification number, dosing regimen, duration of follow-up, and outcome metrics of interest. Risk of bias was evaluated by two researchers according to the RCTs risk of bias assessment tool recommended by the Cochrane Handbook (14), and the following items were deemed as necessary criteria for assessment: selection of the reported result, measurement of the outcome, missing outcome data, deviations from intended interventions, and randomization process. The included studies were sorted into one of the following three categories: low risk, some concerns, and high risk.

### Statistical analysis

After data extraction, statistical analysis was performed using R software (version 4.2.3) and R Studio software. We performed direct comparisons of PD-1/PD-L1 in combination with chemotherapy and chemotherapy alone, and indirect comparisons of PD-L1 + Chemo and PD-1 + Chemo. The primary data analyzed included OS and PFS results expressed as hazard ratio (HR) and 95% confidence interval (CI). Results for the incidence of grade ≥ 3 TRAEs were expressed as risk ratios and its 95% CI. The direct comparisons and the weighting of the literature were visualized further by mapping the network evidence. Network meta-analysis was performed using the JAGS and GEMTC packages, and iterative fitting of the corresponding random-effects or fixed-effects models was performed by constructing a Bayesian Markov Chain-Monte Carlo (MCMC) framework. Non-cumulative probability ranking plots were used to rank the efficacy of different first-line regimens for the treatment of ES-SCLC, and funnel plots were used for publication bias analysis. In this study, The underlying assumption is that the likelihood function is assumed to be a binomial distribution function, the priori probability, since there is no reference value, the gemt analysis package for the R language automatically specifies an uninformative value as the *a priori* probability, and then iteratively corrects the initial theta value by the MCMC framework until the Gelman-Rubin statistic close to 1 indicates convergence, yielding the final effect sizes for the indirect comparisons (15), the posterior probability is the probability that the ‘outcome’ information has been recalibrated, and it is an estimate of the probability that the *a priori* probability has been corrected based on new evidence or information.

## Results

### Characteristics of the included RCTs

**Figure 1:**
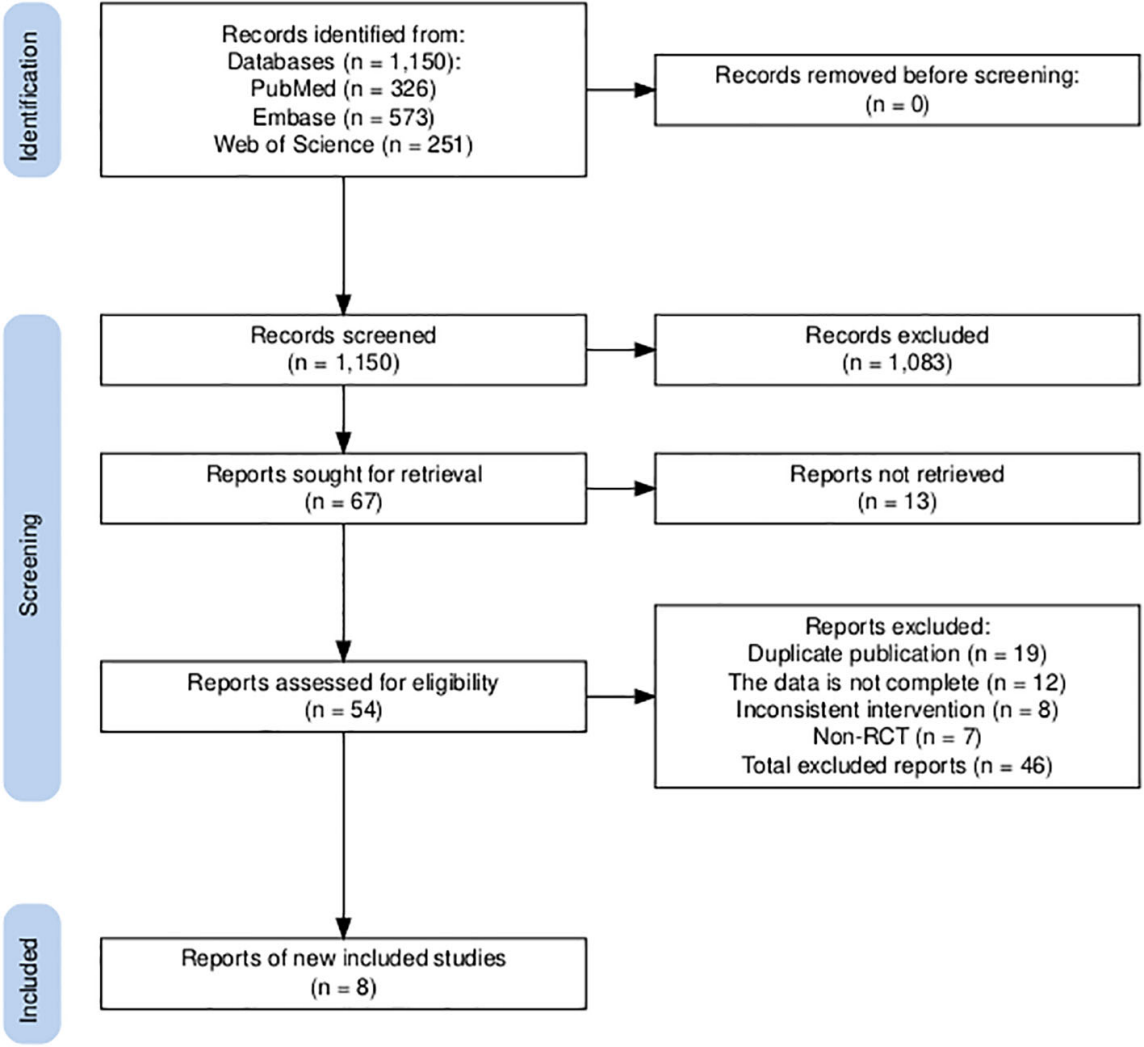
Flow diagram of studies identified, included and excluded.

**Figure 2:**
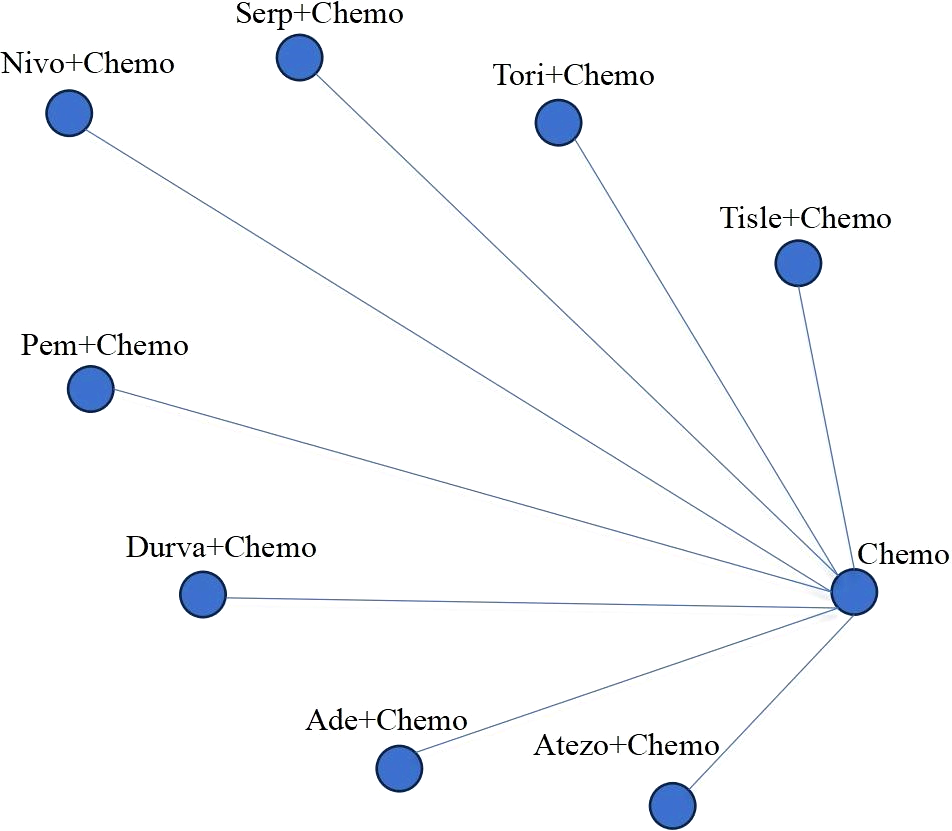
Network of the comparisons. Chemo, chemotherapy; Tisle, tislelizumab; tori, toripalimab; Serp, Serplulimab; Nivo, nivolumab; Pem, pembrolizumab; Durva, durvalumab; Ade, adebrelimab; Atezo, atezolizumab.

An initial literature search identified a total of 1,150 publications. After screening the abstracts and reviewing the full text, a total of eight trials (**Figure 1**), namely, IMpower133, CASPIAN, KEYNOTE-604, EA5161, ASTRUM-005, CAPSTONE-1, RATIONALE-312, and EXTENTORCH, were ultimately included, and a total of 3,559 patients enrolled. Of these, five studies compared the efficacy of PD-1 inhibitors (pembrolizumab, nivolumab, serplulimab, tislelizumab, or toripalimab) in combination with chemotherapy versus chemotherapy alone, and three other studies compared the efficacy of PD-L1 inhibitors (atezolizumab, durvalumab, or adebrelimab) combination chemotherapy versus chemotherapy alone. The network diagram is shown in **Figure 2**. Details of the included RCTs are summarized in **Table 1**.

**Table 1:** Characteristics of patients and outcomes of included trials.

								PD-L1 Subgroups			
Trial name	Treatment	N	OS(m)	HR (os) 95%	PFS(m)	HR (pfs) 95%CI	grade≥3 TRAES%	OS(PD-L1<1%)	OS(PD-L1≥1%)	PFS(PD-L1<1%)	PFS(PD-L1≥1%)
RATIONALE-312	Tislelizumab+Chemo/Chemo	227/230	15.5 VS 13.5	0.75(0.61-0.92)	4.8 vs4.3	0.63(0.51-0.78)	88.5 vs90.0	/	/	/	/
EXTENTORCH	Toripalimab+Chemo/Chemo	223/219	14.6 vs 13.3	0.798(0.648-0.982)	5.8 vs 5.6	0.667(0.539-0.824)	89.6 vs89.4	/	/	/	/
ASTRUM-005	Serplulimab+Chemo/Chemo	389/196	15.8 VS 11.1	0.62(0.50-0.76)	5.8 vs 4.3	0.47(0.38-0.58)	33.2 vs 27.6	HR(OS)0.92(0.44-189)	HR(OS)0.58(0.44-0.77)	/	/
IMpower 133	Atezolizumab+Chemo/Chemo	201/202	12.3 vs 10.3	0.76(0.60-0.95)	5.2 vs 4.3	0.77(0.62-0.96)	58.6/57.6	HR(OS) 0.513(0.297-0.886)	HR(OS) 0.868(0506-1.489)	HR(PFS) 0.523(0.309-0.884	HR(PFS) 0.862(0.509-1.457)
CAPSTONE-1	Adebrelimab+Chem/Chemo	230/232	15.3 vs 12.8	072(0.58-0.90)	5.8 vs 5.6	0.67(0.54-0.83)	37 vs 47	HR(OS)0.66(0.52-0.83)	HR(OS)0.72 (0.33-1.59)	HR(PFS)0.68(0.54-0.85)	HR(PFS)0.70(0.34-1.45
KEYNOTE-604	Pembrolizumab+Chemo/Chemo	288/225	10.8 vs 9.7	0.80(0.64-0.98)	4.5 vs 4.3	0.75(0.61-0.91)	/	HR(OS)0.80(0.58-111)	HR(OS)0.84(0.60-1.18)	HR(PFS) 0.73(0.54-1.01)	HR(PFS) 0.68(0.49-0.94)
CASPIAN	Durvalumab+Chamo/Chemo	268/269	12.9 vs 10.5	0.71(0.60-0.86)	5.1 vs 5.4	0.78(0.645-0.936)	62 vs 62	/	/	/	/
EA5161	Nivolumab+Chemo/Chemo	80/80	11.3 vs 9.3	/	5.5 vs 4.7	0.68(0.48-1.00)	/	/	/	/	/

Chemo, chemotherapy; N, Number; m, months; OS, Overall survival; PFS, Progression-free survival; HR, Hazard ratio; CI, confidence interval; TRAEs, treatment-related adverse events; PD-L1, programmed cell death-ligand 1.

### Risk of bias

We performed a quality assessment according to the criteria of the Cochrane Risk of Bias Tool (2.0), which showed that the risk of attrition bias, reporting bias, performance bias, and selection bias from random sequences was low in the majority of studies (**Supplementary Figure 1**).

### Overall survival

**Figure 3:**
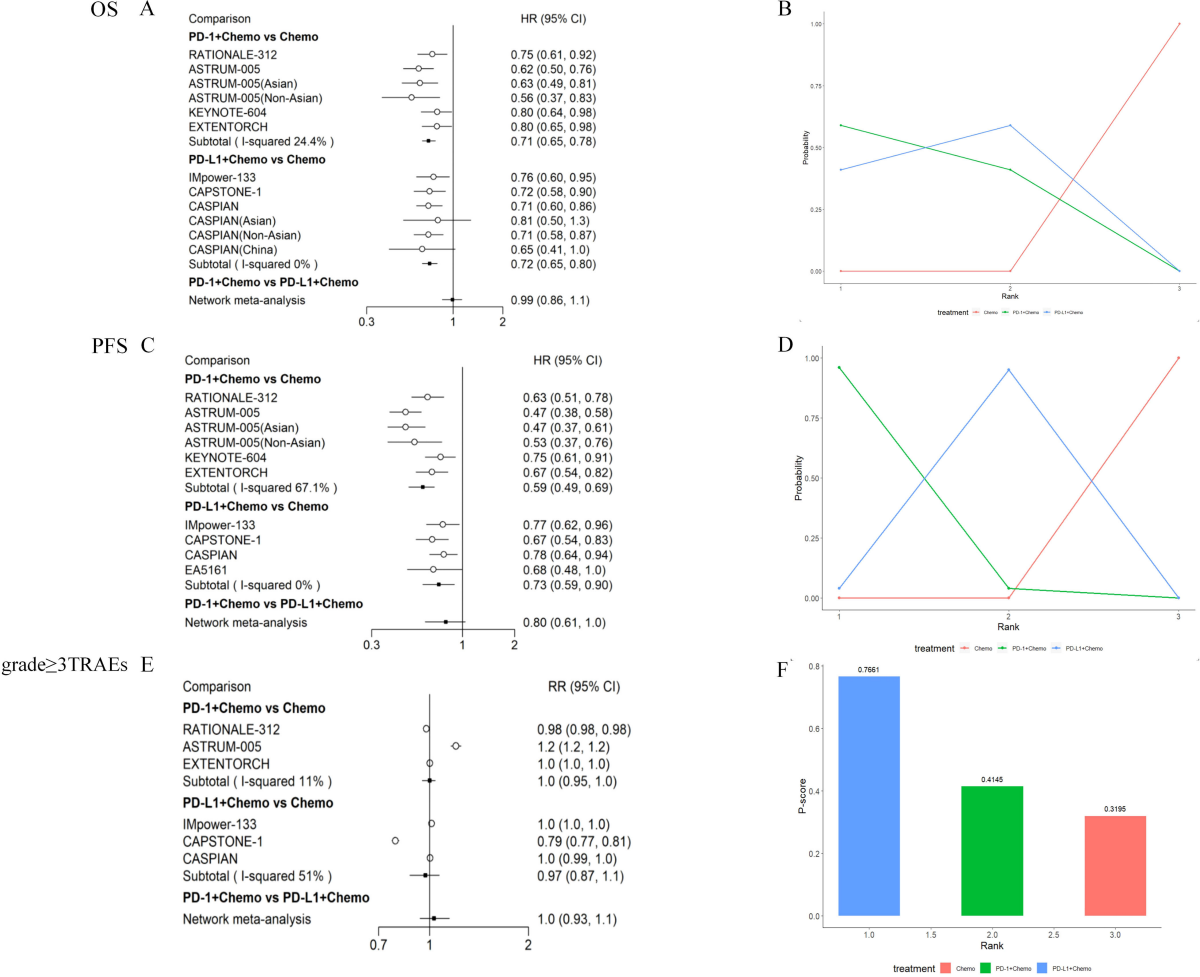
Meta-analysis of PD-1 inhibitor plus chemotherapy (PD-1 + Chemo) or PD-L1 inhibitor plus chemotherapy (PD-L1 + Chemo) compared with chemotherapy alone. Results of direct versus indirect comparisons of **(A)** OS, **(C)** PFS, and **(E)** grade≥ 3 TRAEs in patients with ES-SCLC; patients’ **(B)** OS, **(D)** PFS non-cumulative probability ranking results, and **(F)** P-score ranking results for grade ≥ 3 TRAEs. All of the statistical tests were two-sided. ES-SCLC, extensive-stage small-cell lung cancer; PD-1, programmed cell death 1; PD-L1, programmed cell death-ligand 1; Chemo, chemotherapy; OS, overall survival; PFS, progression-free survival; TRAEs, treatment-related adverse events; HR, hazard ratio; CI, confidence interval; RR, risk ratio.

Direct comparisons showed that OS in ES-SCLC patients was significantly improved by combining PD-1/PD-L1 inhibitors with EP chemotherapy. The HR_PD-1 + Chemo/Chemo_ for those receiving PD-1+ Chemo was 0.71, (95% CI: 0.65-0.78), indicating that PD-1+ Chemo was able to reduce the risk of death in patients by 29%, with a 95% likelihood that the overall mean of the HR values would be between 0.65-0.78, compared to those receiving chemotherapy; and the HR_PD-L1 + Chemo/Chemo_ for those receiving PD-L1+ Chemo was 0.72, (95% CI: 0.65-0.80), suggesting that PD-L1+ Chemo was able to reduce the risk of death in patients by 28%, with a 95% likelihood that the overall mean of the HR values was between 0.65-0.80. Thus, patients who received PD-1+ Chemo/PD-L1+ Chemo had significantly longer survival than those who received chemotherapy alone, with Asian and non-Asian populations sharing the same trends. Indirect comparisons showed no significant difference in OS (HR_PD-1 + Chemo/PD L-1 + Chemo_ = 0.99, 95% CI:0.86–1.1) between PD-1 + Chemo and PD-L1 + Chemo (**Figure 3A**). Ranking analysis based on a non-cumulative probability ranking plot showed that PD-1 + Chemo had a probability of 0.59 to rank first in terms of OS (**Figure 3B**; **Supplementary Table 1**).

### Progression-free survival

PD-1 + Chemo significantly prolonged PFS in patients compared with Chemo alone (HR_PD-1 + Chemo/Chemo_ = 0.59, 95% CI: 0.49–0.69), and the same trend was seen in Asian and non-Asian populations: PD-L1 + Chemo also improving PFS (HR_PD-L1 + Chemo/Chemo_ = 0.73, 95% CI: 0.59–0.90). However, there was no significant difference when comparing PD-1 + Chemo with PD-L1 + Chemo in terms of PFS (HR_PD-1 + Chemo/PD L-1 + Chemo_ = 0.80, 95% CI:0.61-1.0) (**Figure 3C**). The non-cumulative probability ranking plot showed a probability of 0.96 for PD-1 + Chemo to rank first in terms of PFS (**Figure 3D**).

### Toxicity

In the safety analysis, drug toxicity was determined based on the incidence of grade ≥ 3 TRAEs. PD-1 + Chemo did not increase the incidence of grade ≥ 3 TRAEs compared with chemotherapy (HR_PD-1 + Chemo/Chemo_ = 1.0, 95% CI: 0.95–1.0), which was also similar to the results for PD-L1 + Chemo (HR_PD-L1 + Chemo/Chemo_ = 0.97, 95% CI: 0.87–1.1). The incidence of grade ≥ 3 TRAEs was similar for PD-1 + Chemo compared with PD-L1 + Chemo (HR_PD-1 + Chemo/PD L-1 + Chemo_ = 1.0, 95% CI:0.93–1.1) (**Figure 3E**). P-score ranking showed that PD-L1 + Chemo had a P-score of 0.76 and ranked first (**Figure 3F**).

### Subgroup analyses

**Figure 4:**
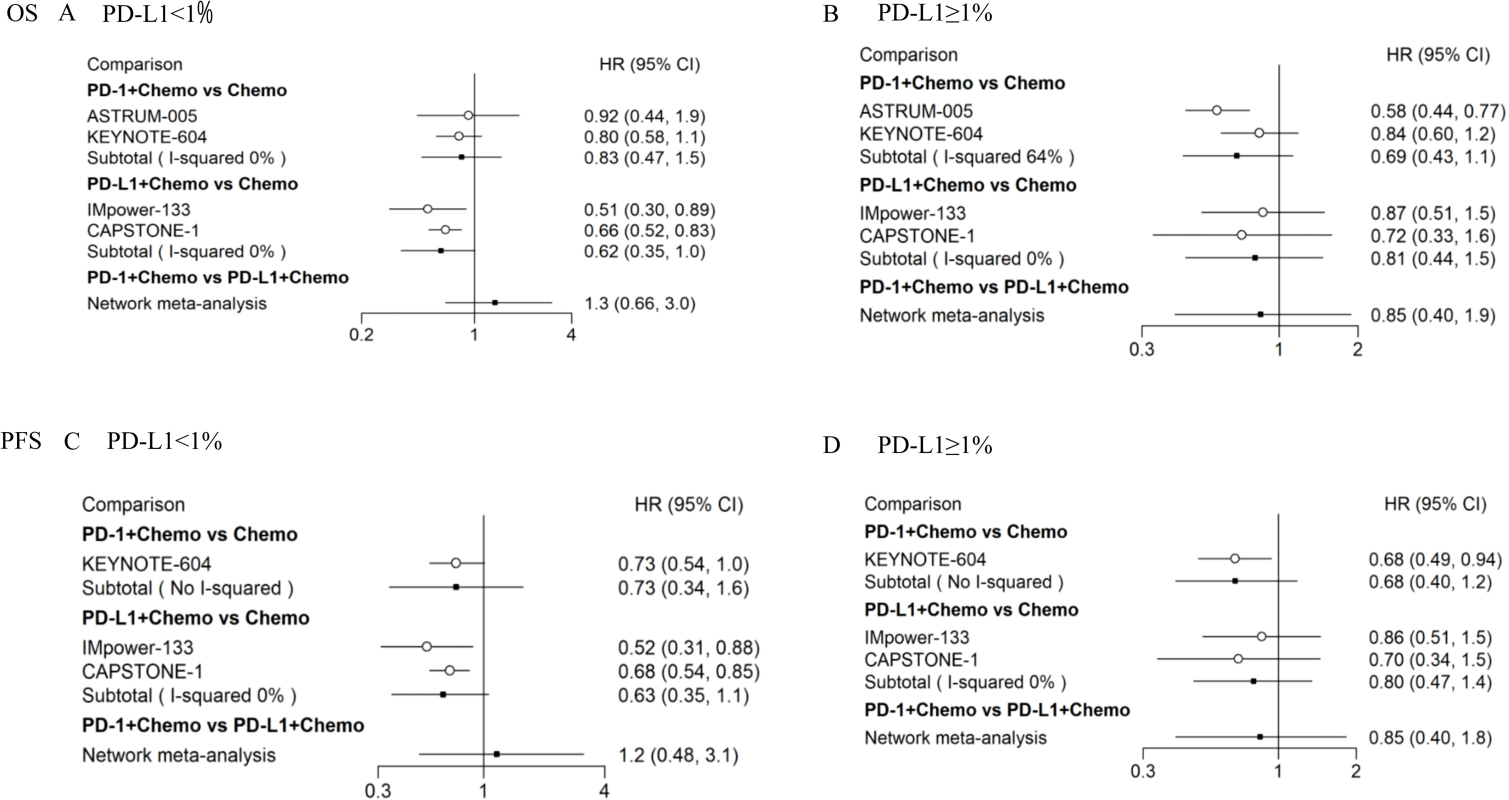
Subgroup analyses of OS and PFS in ES-SCLC patients according to PD-L1 expression levels. Results of direct versus indirect comparison of OS in patients with PD-L1 expression levels < 1% **(A)** and patients with PD-L1 expression levels ≥ 1% **(B)**; results of direct versus indirect comparison of PFS in patients with PD-L1 expression levels < 1% **(C)** and patients with PD-L1 expression levels ≥ 1% **(D)**. All of the statistical tests were two-sided. ES-SCLC, extensive-stage small-cell lung cancer; PD-1, programmed cell death 1; PD-L1, programmed cell death-ligand 1; Chemo, chemotherapy; OS, overall survival; PFS, progression-free survival; HR, hazard ratio; CI, confidence interval.

We performed subgroup analyses based on the level of PD-L1 expression on the tumor cell. For patients with PD-L1 expression levels < 1%, PD-1 + Chemo showed a trend of weakness relative to PD-L1 + Chemo, in terms of OS (HR_PD-1 + Chemo/PD L-1 + Chemo_ = 1.3, 95% CI: 0.66–3.0) (**Figure 4A**), and in terms of PFS (HR_PD-1 + Chemo/PD L-1 + Chemo_ = 1.2, 95% CI: 0.48–3.1) (**Figure 4C**). In contrast, for patients with PD-L1 expression levels ≥ 1%, PD-1 + Chemo showed a trend of superiority relative to PD-L1 + Chemo, in terms of OS (HR_PD-1 + Chemo/PD L-1 + Chemo_ = 0.85, 95% CI:0.40–1.9) (**Figure 4B**), and in terms of PFS (HR_PD-1 + Chemo/PD L-1 + Chemo_ = 0.85, 95% CI:0.40–1.8) (**Figure 4D**).

## Discussion

Platinum-based chemotherapy has been the standard first-line treatment for ES-SCLC since the 1980s. Consolidative chest radiation therapy may reduce the risk of intrathoracic recurrence and promote immune responses (16). Although the incidence of SCLC is slowly declining with the decline in tobacco use, SCLC still remains a difficult cancer to treat (17). Since the PD-L1 inhibitors atezolizumab and durvalumab were approved by the US Food and Drug Administration (FDA) for the first-line treatment of patients with ES-SCLC, the era of immunotherapy for ES-SCLC has begun. Unfortunately, the final results of the studies of pembrolizumab (18) and nivolumab (19, 20) were disappointing. This was followed by positive results for serplulimab and adebrelimab in the first-line treatment of patients with ES-SCLC, and in 2023, the results of the RATIONALE-312 and EXTENTORCH studies revealed the success of tislelizumab and toripalimab in ES-SCLC. These studies raise several questions about the treatment of ES-SCLC, due to the mixed outcomes of PD-1 inhibitors, with both successful and failed studies. Further studies are needed to determine if PD-1 inhibitors are actually effective in ES-SCLC. Additional research is needed to confirm if different ICIs (targeting PD-1 or PD-L1) have the same efficacy and safety in the treatment of ES-SCLC (21).

We conducted an updated meta-analysis to summarize currently published or updated data and to provide a comprehensive assessment. As direct comparative studies are unlikely, our study attempted to indirectly compare the efficacy of PD-1 + Chemo and PD-L1 + Chemo. To the best of our knowledge, this is the most up-to-date and comprehensive meta-analysis, including the largest number of clinical studies cases.

Based on a comprehensive review of current RCTs, we included eight RCTs involving 3,559 patients. The findings showed that compared with chemotherapy alone, both PD-1 + Chemo and PD-L1 + Chemo had statistically meaningful differences in terms of OS and PFS, which suggests that PD-1 + Chemo improves outcomes in ES-SCLC. One study that included 1,553 patients also demonstrated a significant improvement in OS in patients with ES-SCLC with the addition of PD-1 and PD-L1 ICIs (HR: 0.76; 95% CI: 0.68–0.85) (22). The present study enriched the research data on PD-1 inhibitors in the first-line treatment of ES-SCLC after the inclusion of the results of tislelizumab and toripalimab, but PD-1 + Chemo versus PD-L1 + Chemo did not produce a significant improvement in OS and PFS. The non-cumulative probability ranking results suggest that PD-1 + Chemo ranks highest in terms of efficacy, which may be related to the immune microenvironment of SCLC, where PD-L1 inhibitors can only inhibit the binding of PD-1 to PD-L1, and the tumor cells may evade the anti-tumor immune response through the binding of PD-L2 and PD-1 (23, 24). Previous studies have found that PD-1 inhibitors block both recognition and binding between PD-1 and its ligands (PD-L1 and PD-L2), and thus PD-1 may result in a higher incidence of adverse events (25, 26). Regarding safety, no significant differences were found in the incidence of grade ≥ 3 TRAEs between PD-1 + Chemo and PD-L1 + Chemo compared with PD-L1 + Chemo.

Previous studies have shown that the expression level of PD-L1 is a potential biomarker for predicting the response to ICIs in various cancers (27, 28); however, the role of the expression level of PD-L1 in SCLC in predicting the efficacy of ICIs is not obvious (29). The reason for this is unclear, but it may be due to the fact that in SCLC, PD-L1 is predominantly expressed on the surface of tumor-infiltrating immune cells rather than on the surface of tumor cells, which can independently attenuate anti-cancer immunity (30, 31).

Compared to other meta-analysis techniques, Bayesian methods are able to combine *a priori* information and data to deal with uncertainty in a natural way, and this approach is particularly effective in the face of uncertainty and complex data structures. The inferences provided by Bayesian methods also incorporate prior knowledge, so the results are often more interpretable. Bayesian methods are able to handle complex nonlinear relationships and provide richer information by learning probability distributions, providing greater flexibility. However, Bayesian methods have higher computational complexity, which increases computational cost and time, and also the subjective probabilities used in Bayesian methods can be controversial, especially in areas where consensus is lacking (32).

In this study, we performed subgroup analyses according to the different expression levels of PD-L1. PD-1 + Chemo showed a trend toward a weaker survival rate in patients with a PD-L1 expression level of < 1% compared with PD- L1 + Chemo, while a trend toward a higher survival rate in patients with a PD- L1 expression level of ≥ 1% was evident. However, this result needs to be interpreted with caution and should not guide clinicians to select appropriate ICIs based on PD-L1 expression levels.

There are several limitations of this study that should be noted. First, the study included eight RCTs, three of which were conference abstracts, which could lead to potential bias. Second, differences in treatment regimens and baseline characteristics of the population may have contributed to the heterogeneity of results. Lastly, the impact of PD-1/PD-L1 + Chemo on other meaningful endpoints, such as quality of life, was not further assessed. Therefore, future studies with larger sample sizes and prospective clinical trials are still needed to validate these findings.

In summary, the study results study suggest that there is no statistically significant difference in OS, PFS, and grade ≥ 3 TRAEs with PD-1 + Chemo or PD-L1 + Chemo for first-line treatment in patients with ES-SCLC.

## Data Availability

The original contributions presented in the study are included in the article/**Supplementary Material**. Further inquiries can be directed to the corresponding author.
