# Supplementary material for: Updated Bayesian network meta-analysis on the efficacy and safety of PD−1 versus PD−L1 inhibitors in first−line treatment with chemotherapy for extensive−stage small-cell lung cancer
Source: Front Oncol. 2025 Jan 28;14:1455306. doi: 10.3389/fonc.2024.1455306 (PMC11810729; doi:10.3389/fonc.2024.1455306)
Supplement: Supplementary file 1 [file DataSheet1.docx]

**Updated Bayesian network meta-analysis on the efficacy and safety of PD‑1 versus PD‑L1 inhibitors in first‑line treatment with chemotherapy for extensive‑stage small-cell lung cancer**

Ke Wang^1,2†^, Chuangjie Zheng^1,2†^, Xinrong Chen^1,2^, Penghui Lin^3^, Mengge Lin^3^, Cuizhen Chen^3^, and Linzhu Zhai^3^*

**Supplementary Material**

| Treatment | Ranks (OS) | Probability (OS) | Rank (PFS) | Probability (PFS) |
| --- | --- | --- | --- | --- |
| Chemo | first | 0.00 | first | 0.00 |
| Chemo | second | 0.00 | second | 0.00 |
| Chemo | third | 1.00 | third | 1.00 |
| PD-1+Chemo | first | 0.59 | first | 0.96 |
| PD-1+Chemo | second | 0.41 | second | 0.04 |
| PD-1+Chemo | third | 0.00 | third | 0.00 |
| PD-L1+Chemo | first | 0.41 | first | 0.04 |
| PD-L1+Chemo | second | 0.59 | second | 0.96 |
| PD-L1+Chemo | third | 0.00 | third | 0.00 |

Supplementary Table 1. Non-cumulative probability ranking analysis results.

Abbreviations: Chemo, chemotherapy; OS, Overall survival; PFS, Progression-free survival; PD-1, programmed cell death 1; PD-L1, programmed cell death-ligand 1.

**Search strategies for Web of Science, Embase and PubMed.**

Web of Science

TS=("small cell lung cancer" OR "SCLC" OR "Small Cell Lung Carcinoma" NOT "non-small cell lung cancer") AND TS=("immunotherapy" OR "immune checkpoint inhibitor" OR "ipilimumab" OR "serplulimab" OR "nivolumab" OR "pembrolizumab" OR "atezolizumab" OR "durvalumab" OR "toripalimab" OR "adebrelimab" OR "tislelizumab" OR "PD-1" OR "programmed cell death 1 receptor" OR "PD-L1" OR "programmed death-ligand 1" OR "CTLA-4" OR "cytotoxic T-lymphocyte-associated protein 4") AND TS=("Extensive-stage" OR "Extensive stage" OR "Extensive-disease" OR "Extensive disease" OR "advanced" OR "extensive")

Embase

('sclc':ab,ti OR 'small cell lung cancer':ab,ti OR 'small cell lung carcinoma':ab,ti) NOT ('non-small cell lung carcinoma':ab,ti OR 'non-small cell lung cancer':ab,ti OR 'nsclc':ab,ti) AND ('immunotherapy':ab,ti OR 'immune checkpoint inhibitor':ab,ti OR 'ipilimumab':ab,ti OR 'serplulimab':ab,ti OR 'nivolumab':ab,ti OR 'pembrolizumab':ab,ti OR 'atezolizumab':ab,ti OR 'durvalumab':ab,ti OR 'toripalimab':ab,ti OR 'adebrelimab':ab,ti OR 'tislelizumab':ab,ti OR 'pd-1':ab,ti OR 'programmed cell death 1 receptor':ab,ti OR 'pd-l1':ab,ti OR 'programmed death-ligand 1':ab,ti OR 'ctla-4':ab,ti OR 'cytotoxic t-lymphocyte-associated protein 4':ab,ti) AND ('extensive-stage':ab,ti OR 'extensive stage':ab,ti OR 'extensive-disease':ab,ti OR 'extensive disease':ab,ti OR 'advanced':ab,ti OR 'extensive':ab,ti)

PubMed

((((((((((((((CTLA-4[Title/Abstract]) OR (cytotoxic T-lymphocyte-associated protein 4[Title/Abstract])) OR (PD-1[Title/Abstract])) OR (programmed death receptor 1[Title/Abstract])) OR (PD-L1[Title/Abstract])) OR (programmed death-ligand 1[Title/Abstract])) OR (immune checkpoint inhibitor[Title/Abstract])) OR (ipilimumab[Title/Abstract])) OR (serplulimab[Title/Abstract])) OR (nivolumab[Title/Abstract])) OR (pembrolizumab[Title/Abstract])) OR (atezolizumab[Title/Abstract])) OR (durvalumab[Title/Abstract])) OR (toripalimab[Title/Abstract])) OR (adebrelimab[Title/Abstract])) OR (tislelizumab[Title/Abstract])).


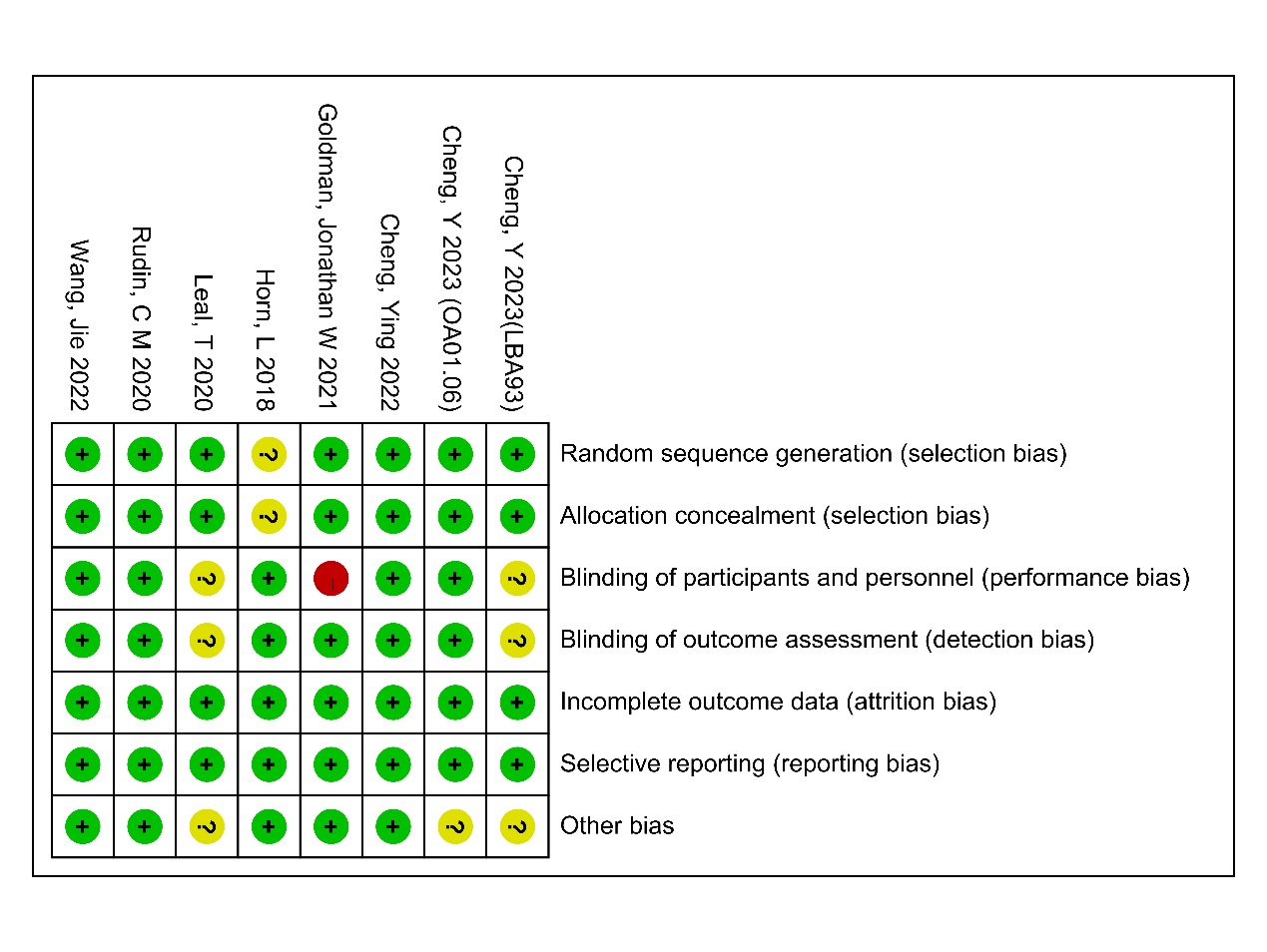


Supplementary Figure 1. Summary of results from assessment of studies using the Cochrane risk of

bias tool. one study exhibited high risks of performance bias, the risks of attrition bias, reporting bias, performance bias and selection bias for random sequence generation was low in most of the studies.


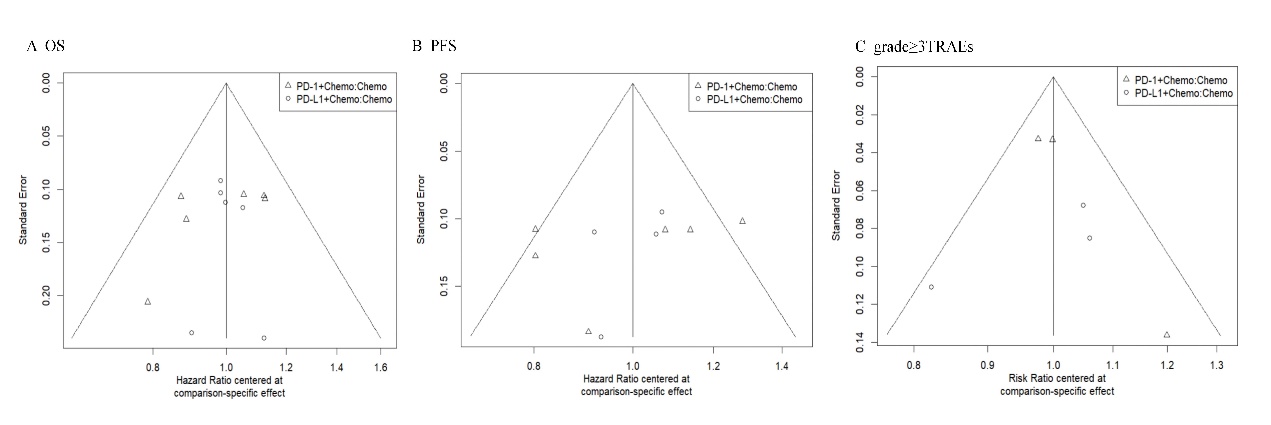


Supplementary Figure 2. Funnel plot of publication bias analysis for (A) overall survival (B) progression-free survival, and(C) grade ⩾3 treatment-related adverse events.

1.
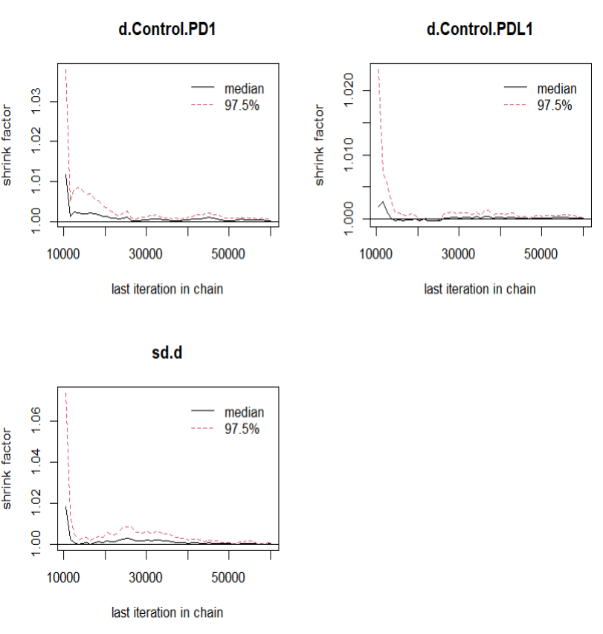

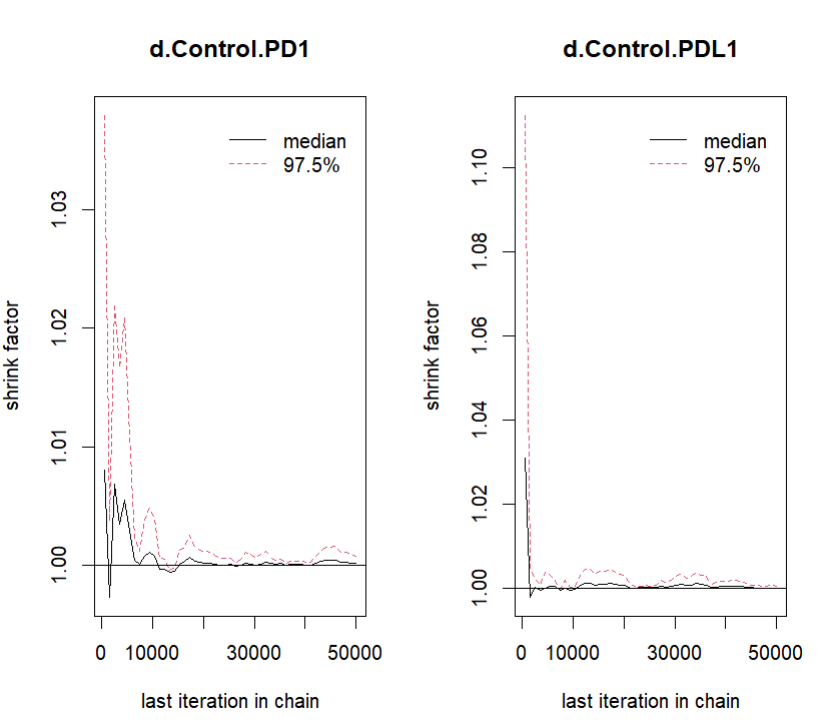
 (B)

Supplementary Figure 3. Convergence diagnostic visualization analysis results. (A) Overall survival (indirect comparison), (B) Progression-free survival (indirect comparison)

(A)


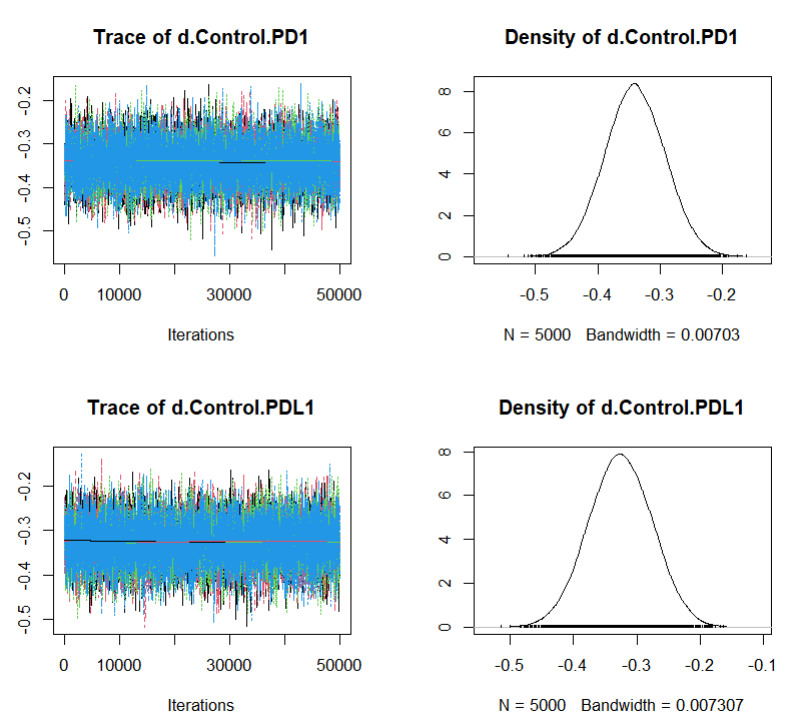


(B)


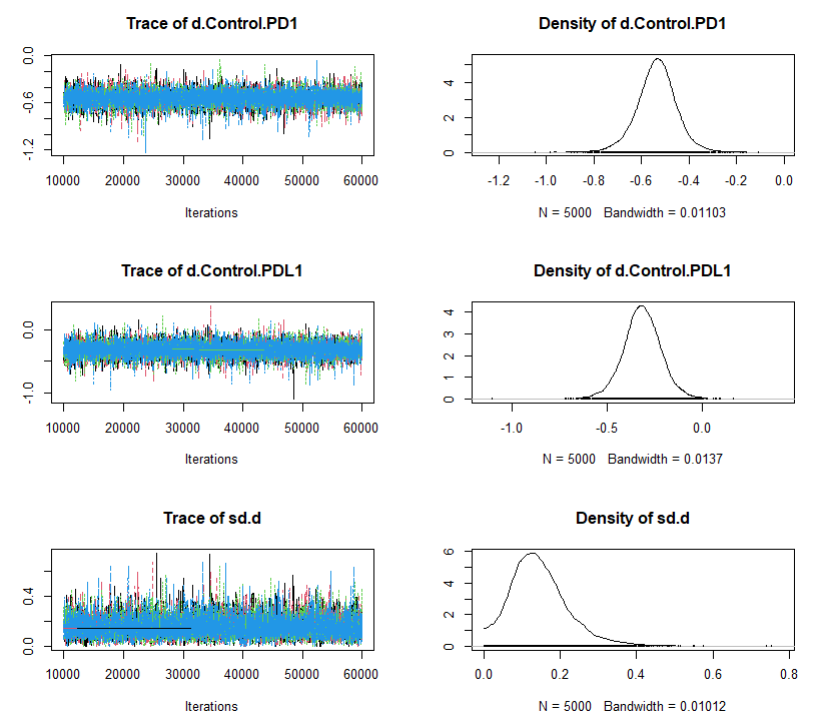


Supplementary Figure 4. Determine model convergence by examining dynamic trajectory plots and density plots. (A) Overall survival (indirect comparison) (B) Progression-free survival (indirect comparison).
